# Automatically Annotated Dataset of a Ground Mobile Robot in Natural Environments via Gazebo Simulations

**DOI:** 10.3390/s22155599

**Published:** 2022-07-26

**Authors:** Manuel Sánchez, Jesús Morales, Jorge L. Martínez, J. J. Fernández-Lozano, Alfonso García-Cerezo

**Affiliations:** Robotics and Mechatronics Lab, Andalucía Tech, Universidad de Málaga, 29071 Málaga, Spain; manuel.sanchez.m@uma.es (M.S.); jesus.morales@uma.es (J.M.); jfl@uma.es (J.J.F.-L.); ajgarcia@uma.es (A.G.-C.)

**Keywords:** synthetic dataset, Gazebo simulator, UGV navigation, natural environments, automatic data labeling, 3D LiDAR, stereo camera

## Abstract

This paper presents a new synthetic dataset obtained from Gazebo simulations of an Unmanned Ground Vehicle (UGV) moving on different natural environments. To this end, a Husky mobile robot equipped with a tridimensional (3D) Light Detection and Ranging (LiDAR) sensor, a stereo camera, a Global Navigation Satellite System (GNSS) receiver, an Inertial Measurement Unit (IMU) and wheel tachometers has followed several paths using the Robot Operating System (ROS). Both points from LiDAR scans and pixels from camera images, have been automatically labeled into their corresponding object class. For this purpose, unique reflectivity values and flat colors have been assigned to each object present in the modeled environments. As a result, a public dataset, which also includes 3D pose ground-truth, is provided as ROS bag files and as human-readable data. Potential applications include supervised learning and benchmarking for UGV navigation on natural environments. Moreover, to allow researchers to easily modify the dataset or to directly use the simulations, the required code has also been released.

## 1. Introduction

Context-aware navigation of an Unmanned Ground Vehicle (UGV) in unstructured environments commonly adopts learning-based methods to interpret onboard sensor data [1]. Thus, the availability of public datasets has become very relevant for developing, training, evaluating, and comparing new techniques on artificial intelligence [2]. In particular, deep learning methods require large datasets with representative data as input [3].

Most datasets for UGVs on outdoor environments offer real tridimensional (3D) data for autonomous driving on roadways [4,5], but also for Simultaneous Localization and Mapping (SLAM) [6,7], direct motion control [8], precision agriculture [9,10], planetary exploration [11,12] or Search and Rescue (SAR) [13]. These datasets usually provide the vehicle pose ground-truth, and, increasingly, they also include tagged exteroceptive data from range and image sensors [14,15].

Semantic annotation of outdoor raw data is usually performed manually [3,15,16]. However, this is a time-consuming, difficult and error-prone process [17]. To speed it up, specific software tools can be employed to assist humans while tagging picture pixels or 3D scan points interactively [18,19].

Generating synthetic annotated data is an alternative to manual or assisted tagging that can be automated completely [20]. Large-scale virtual environments offer the opportunity to closely replicate portions of the real world [21,22]. For this purpose, the research community has explored the use of video games [23,24], procedural generation [25,26] and robotic simulations [27,28] to obtain realistic sensor data.

Furthermore, UGV simulations allow to test autonomous navigation safely [29,30]. In this way, highly controlled and repeatable experiments can be performed, which are especially relevant for reinforcement learning [22].

Up to the knowledge of the authors, there are no publicly available datasets with tagged data obtained from a UGV navigating in varied natural settings. This issue can be mainly due to the inherent complex characteristics of such environments, such as terrain roughness, diverse vegetation or low structuring. In particular, the main objective of this paper is to try to mitigate this gap.

Specifically, the paper presents a new synthetic dataset that has been generated with the robotic simulator Gazebo [31] using the realistic physical engine ODE (Open Dynamics Engine). Gazebo is an open-source and high-fidelity robot emulator for indoor [32] and outdoor environments [29]. In addition, the open-source Robot Operating System (ROS) [33] has been integrated into Gazebo to mimic the same software of the mobile robot [28]. ROS also provides tools to easily record and replay sensor data using bag files [7].

For our dataset (https://www.uma.es/robotics-and-mechatronics/info/132852/negs-ugv-dataset, accessed on 22 July 2022), a Husky mobile robot has followed several paths on four natural environments using ROS. The data is shared in the form of ROS bags that contain synchronized readings from several sensors: wheel tachometers, an inertial measurement unit (IMU), a Global Navigation Satellite System (GNSS) receiver, a stereo camera, and a 3D Light Detection and Ranging (LiDAR) scanner. The ground-truth 3D pose of the vehicle as well as labeled LiDAR points and tagged camera pixels are also fully available. Moreover, to easily modify the natural environments, the navigation conditions or the sensor equipment, all the required software to generate the dataset has also been released in the website.

In this way, two original contributions can be highlighted for this paper:

- A new dataset obtained from realistic Gazebo simulations of a UGV moving on natural environments is presented.
- The released dataset contains 3D point clouds and images that have been automatically annotated without errors.

We believe that this labeled dataset can be useful for training data-hungry deep learning techniques such as image segmentation [34] or 3D point cloud semantic classification [35], but it can also be employed for testing SLAM [36] or for camera and LiDAR integration [37]. Furthermore, the robotic simulations can be directly employed for reinforcement learning [38].

The remainder of the paper is organized as follows. The next section describes the modeling of the ground mobile robot and of the natural environments in Gazebo. Section 3 presents the simulated experiments that have been carried out to generate the data. Section 4 shows how LiDAR and camera data have been automatically labeled. Then, Section 5 describes the dataset structure and the supplementary material. Finally, the last section draws conclusions and suggests some future work.

## 2. Gazebo Modeling

### 2.1. Husky Mobile Robot

**Figure 1:**
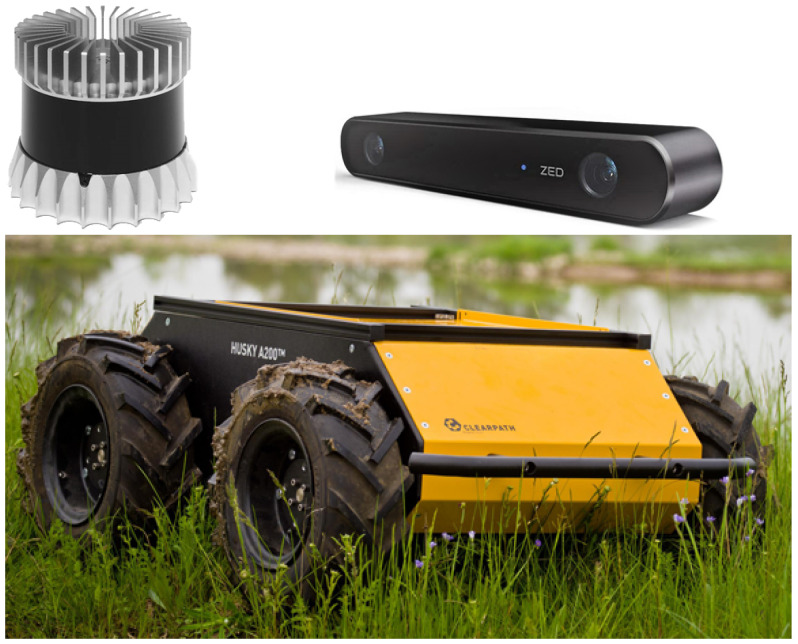
Photographs of an Ouster OS1-64 LiDAR (**up-left**), a ZED-2 stereo camera (**up-right**) and a Clearpath Husky UGV (**down**).

Husky is a popular commercial UGV (see Figure 1) from Clearpath Robotics (https://clearpathrobotics.com/husky-unmanned-ground-vehicle-robot/, accessed on 22 July 2022) that employs the well-known ROS programming environment. It is a 50 kg four-wheeled skid-steered mobile robot with high torque drive for outdoor navigation at a maximum speed of 1 m/s. Its external dimensions are 0.99 m long, 0.67 m width and 0.39 m high.

**Figure 2:**
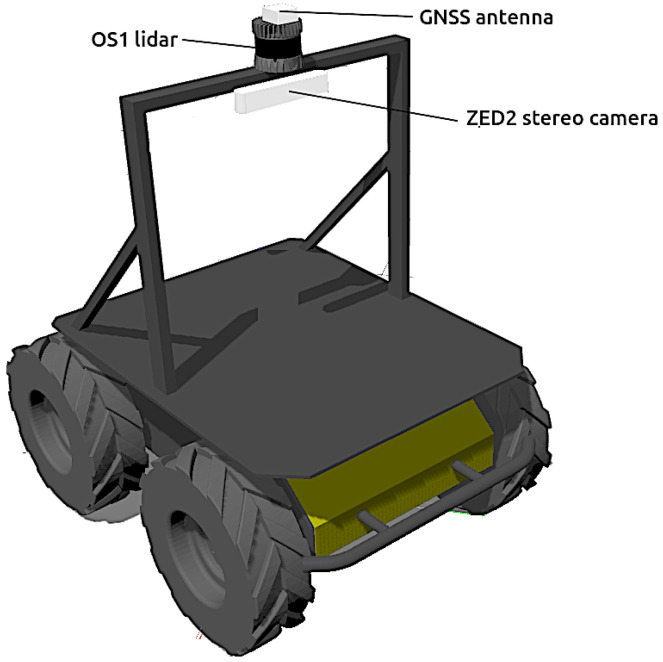
Model of Husky in Gazebo with a stand for its exteroceptive sensors.

Husky can be simulated in Gazebo using the husky_gazebo packages (https://github.com/husky/husky_simulator, accessed on 22 July 2022) provided by the manufacturer (see Figure 2). In addition, a complete set of sensors for context-aware navigation has been mounted onboard:

**Tachometers.**  A Gazebo plugin reads the angular speed of each wheel and publishes it in an ROS topic at a rate of 10 Hz.
**IMU.**  A generic IMU has been included inside the robot to provide its linear accelerations, angular velocities and 3D attitude. The data, composed of nine values in total, is generated directly from the physics engine ODE during the simulations with an output rate of 50 Hz.
**GNSS.**  The antenna of a generic GNSS receiver is incorporated on top of the vehicle (see Figure 2). The latitude λ, longitude ϕ and height *h* coordinates are directly calculated from the Gazebo simulation state at a rate of 2 Hz.
**Stereo camera.**  The popular ZED-2 stereo camera (https://www.stereolabs.com/assets/datasheets/zed2-camera-datasheet.pdf, accessed on 22 July 2022), with a baseline of 0.12 m, have been chosen (see Figure 1). The corresponding Gazebo model has been mounted centered on a stand above the robot (see Figure 2). The main characteristic of this sensor can be found in Table 1.
**3D LiDAR.**  The selected 3D LiDAR is an Ouster OS1-64 (https://ouster.com/products/os1-lidar-sensor/, accessed on 22 July 2022)), which is a small high-performance multi-beam sensor (see Table 1) with an affordable cost (see Figure 1). It is also mounted on top of the stand to increase environment visibility (see Figure 2).

**Figure 3:**
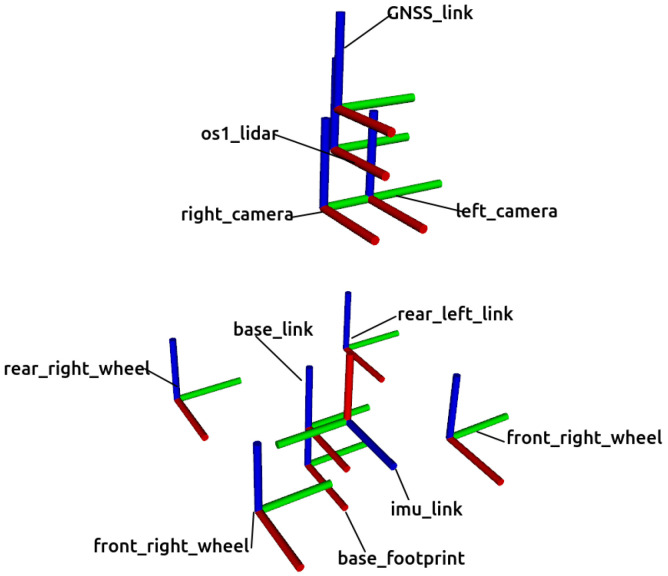
Reference frames employed in the Gazebo model of Husky. Their *X*, *Y* and *Z* axes are represented in red, green and blue colors, respectively.

All the reference frames employed for this mobile robot are represented in Figure 3. The coordinate system base_footprint is placed at the center of the contact points of the four wheels with the ground with its local *X*, *Y* and *Z* axes pointing forward, to the left and upwards, respectively. The main reference frame of Husky is called base_link and is placed 0.13228 m above base_footprint with the same axes orientation. There are also coordinate systems for every sensor, whose pose with respect to base_link can be found in Table 2.

### 2.2. Natural Environments

Four different natural settings have been modeled in Gazebo realistically. Each environment, which is contained in a rectangle 50 m wide and 100 m long, has distinct features as discussed below.

The global reference system for Gazebo is placed at the center of each rectangle, where its *X* and *Y* axes coincide with the longest and shorter symmetry lines, respectively. For the GNSS receiver, this center corresponds to the following geodetic coordinates: $\lambda =49.9{}^{\circ }$, $\phi =8.9{}^{\circ }$ and h=0 m, where *X* points to the North, *Y* to the West and *Z* upwards.

#### 2.2.1. Urban Park

**Figure 4:**
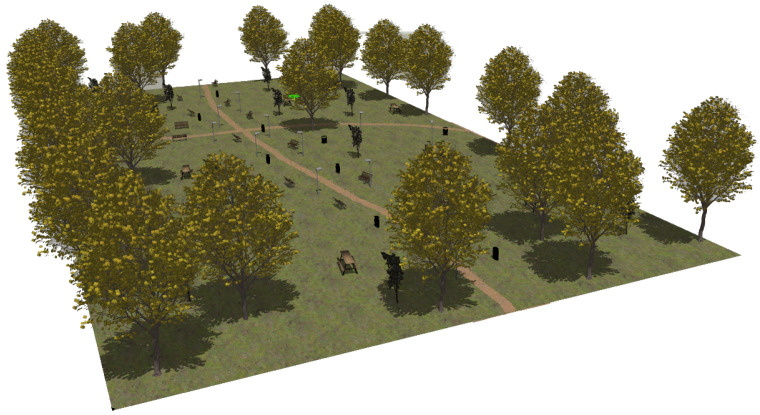
The urban park environment modeled in Gazebo.

The first modeled surroundings is an urban park (see Figure 4). The almost plane ground contains two trails for pedestrians. Apart from natural elements such as trees and bushes, it also includes the following artificial objects: lamp posts, benches, tables and rubbish bins.

#### 2.2.2. Lake’s Shore

**Figure 5:**
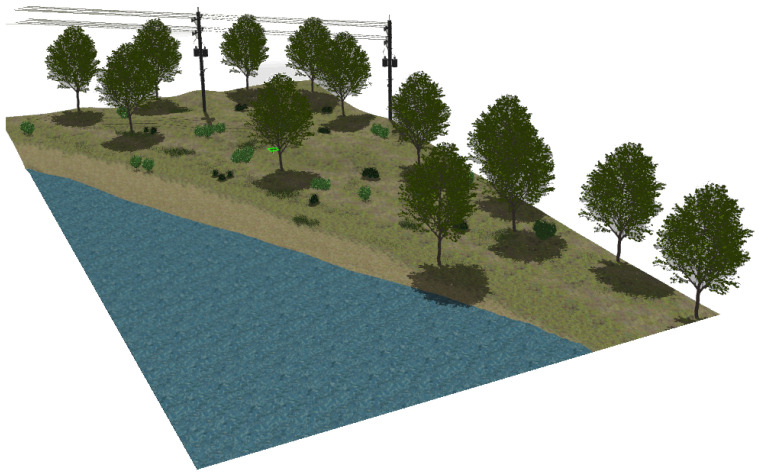
The lake’s shore environment modeled in Gazebo.

The second natural environment contains a lake, its shore, high grass and different kinds of bushes and trees (see Figure 5). The terrain is elevated above the lake a few meters and includes two electrical power poles with their corresponding aerial wires.

#### 2.2.3. Dense Forest

**Figure 6:**
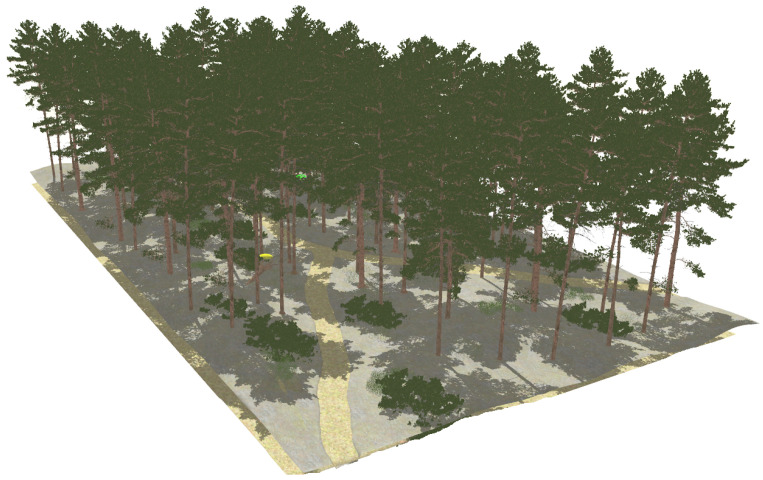
The dense forest environment modeled in Gazebo.

The third modeled surroundings consists of a high density forest crossed by two trails (see Figure 6). The uneven terrain is populated with high grass, stones, bushes, trees and fallen trunks.

#### 2.2.4. Rugged Hillside

**Figure 7:**
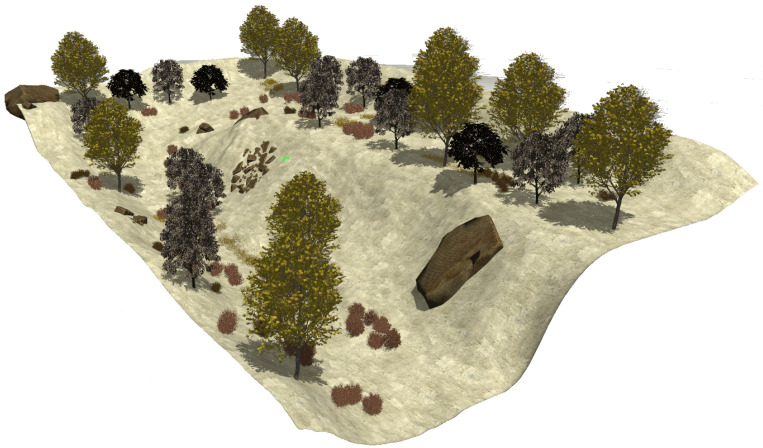
The rugged hillside environment modeled in Gazebo.

The fourth natural setting represents the hillside of a mountain (see Figure 7). This dry and rocky environment contains steep slopes with sparse vegetation composed of high grass, bushes and trees.

## 3. Simulated Experiments

The ROS programming environment has been integrated in Gazebo by using ROS messages and services, and with Gazebo plugins for sensor output and motor input.

Autonomous navigation of the Husky mobile robot has been simulated while following two paths on each of the previously presented environments. The data is recorded in the form of ROS bags that contain synchronized readings from all the onboard sensors. Virtual measurements from all the sensors have been acquired free of noise. If required, an appropriate type of noise for each sensor can be easily added to the recorded data later.

Navigation on natural terrains has been implemented by following way-points given by their geodetic coordinates. The ordered list of way-points broadly represents the trajectory that the vehicle should follow to safely avoid obstacles. Way-points have been manually selected by taking into account the limited available traversable space in each environment. Thus, the separation between consecutive way-points can vary between 5 m and 20 m depending on the proximity to obstacles.

The UGV is commanded with a constant linear velocity of 0.3 m/s. The angular velocity is automatically selected to head the robot towards the current goal. When the Husky approaches less than 2 m, the next way-point from the list is selected. Finally, when arriving at the last goal, the mobile robot is stopped.

The ROS controller, which is assumed to be performed entirely by the Husky computer, only employs planar coordinates from the onboard sensors, i.e., latitude and longitude from GNSS and absolute heading from the IMU. It adjusts the angular speed of the UGV with a proportional value of the heading error of the vehicle with respect to the current goal [30].

**Figure 8:**
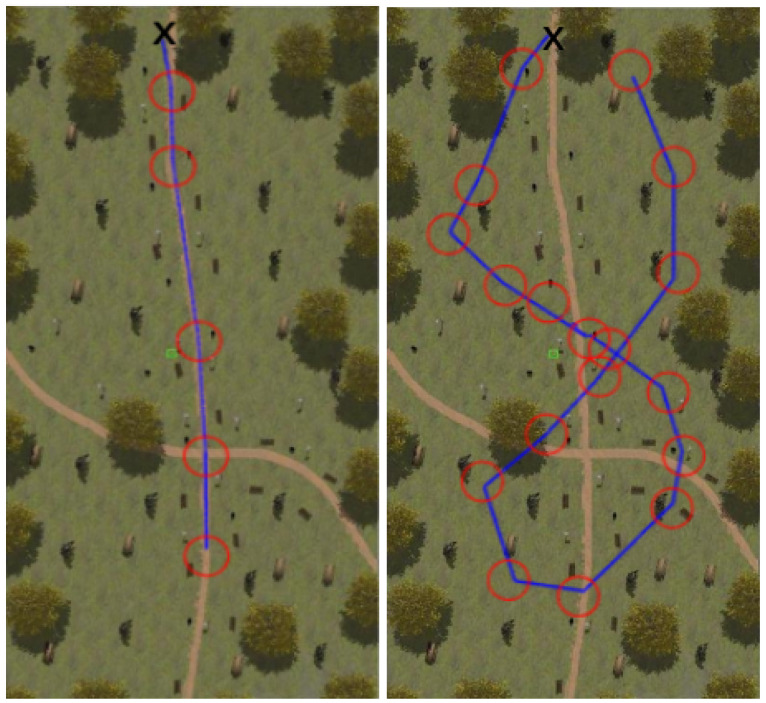
Paths followed during data acquisition in the park environment.

**Figure 9:**
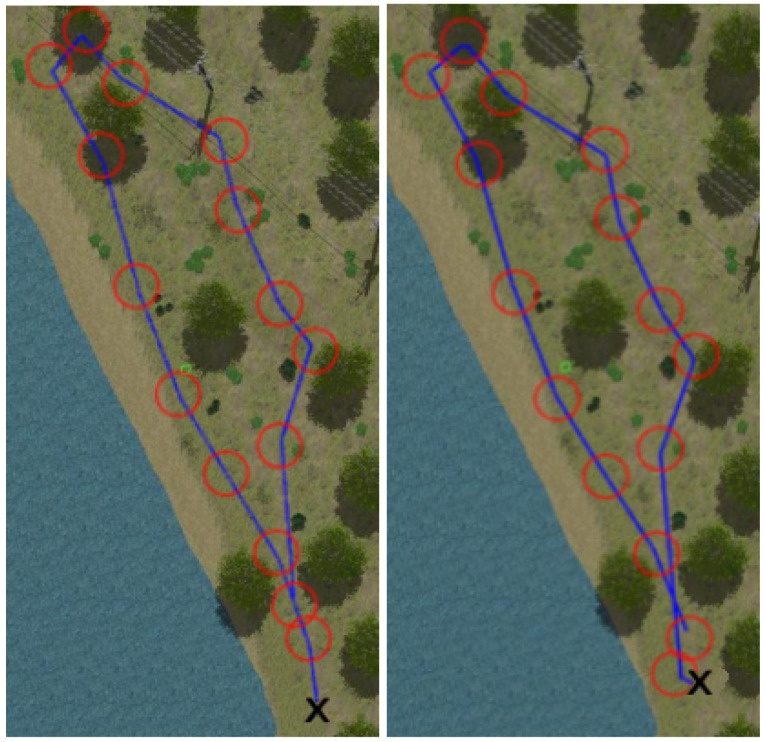
Paths followed during data acquisition in the lake environment.

**Figure 10:**
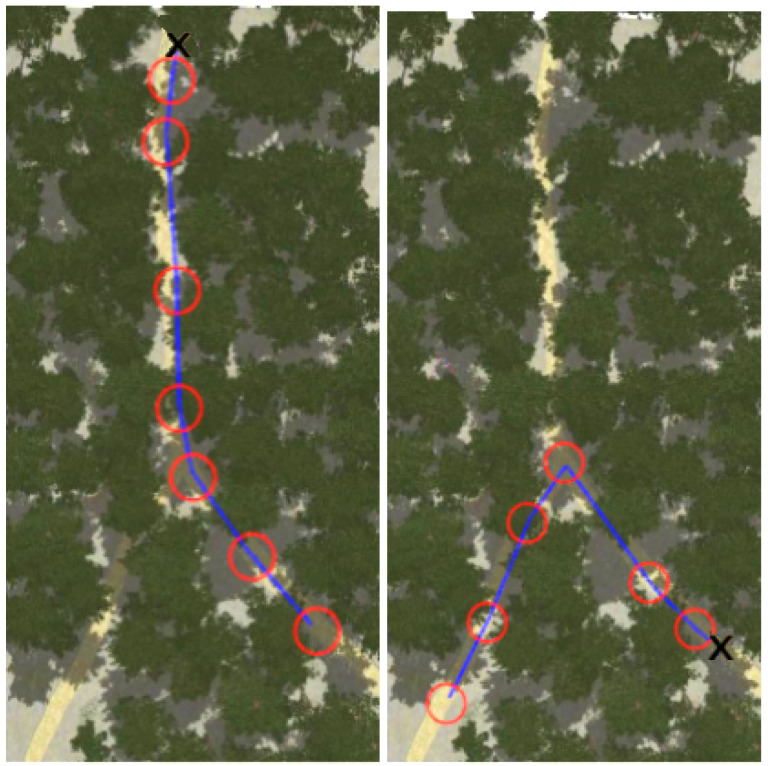
Paths followed during data acquisition in the forest environment.

**Figure 11:**
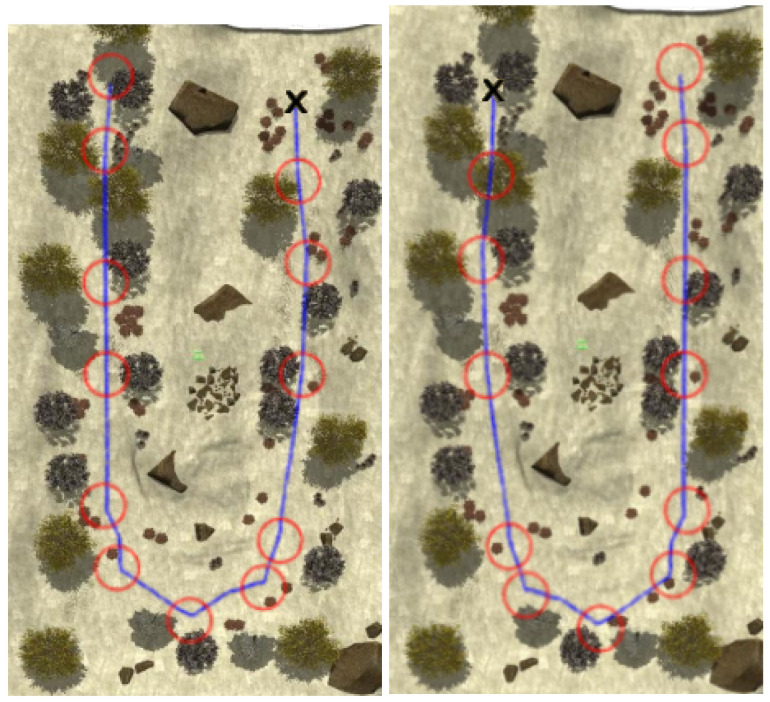
Paths followed during data acquisition in the hillside environment.

Figure 8, Figure 9, Figure 10 and Figure 11 show aerial views of the two paths followed by Husky on each environment with blue lines. Different paths can be observed for the park and forest environments, but they are very similar for the lake’s shore and the hillside, where paths have been tracked in opposite directions. In all these figures, red circles with a radius of 3 m indicate the way-points and the ’x’ letter marks the UGV initial position.

## 4. Automatic Tagging

To automatically annotate objects to 3D LiDAR points, arbitrary reflectivity values have been assigned to each object in the Gazebo collision model with the exception of the sky and water (see Table 3). These two elements do not produce any range at all: in the first case because it cannot be sensed with LiDAR and in the second case to emulate laser beams that are deflected by water surface.

**Figure 12:**
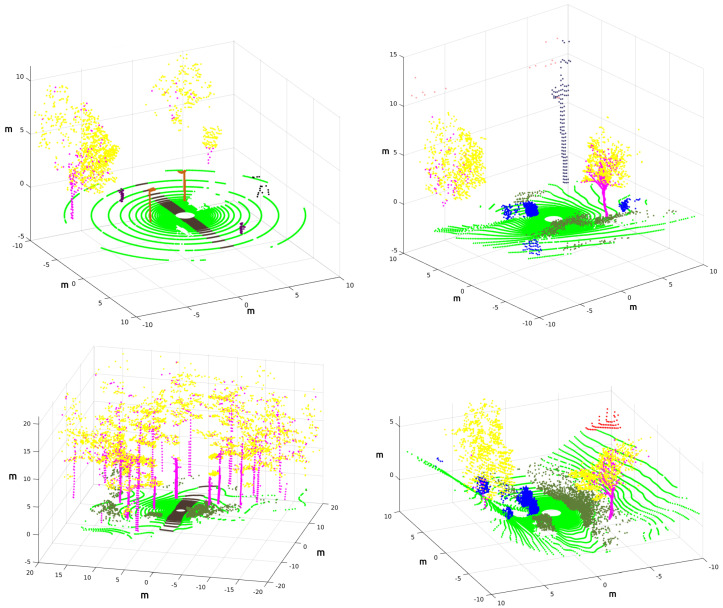
Annotated point clouds for the park (**up-left**), the lake (**up-right**), the forest (**left-down**) and the hill (**right-down**). Refer to Table 3 for element colors.

Thus, the returned intensity values of each laser ray in Gazebo can be used to tag every 3D point with its corresponding object without errors [17]. Figure 12 shows examples of 3D point clouds completely annotated using this procedure, where the coordinates refer to the os1_lidar frame from which each 3D point cloud was acquired. The Red-Green-Blue (RGB) colors contained in Table 3 are employed to distinguish different elements.

The procedure to automatically tag each pixel requires post-processing after simulation because images from the stereo camera are not captured during navigation. To proceed with it, two different kinds of visual models in Gazebo are used: realistic and plain.

**Figure 13:**
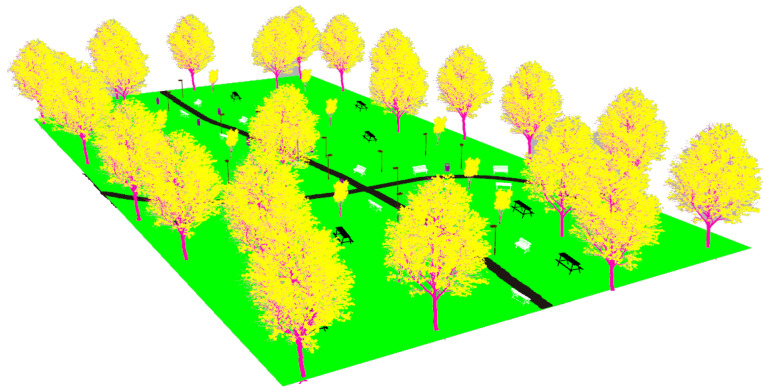
The plain visual model of the park environment.

**Figure 14:**
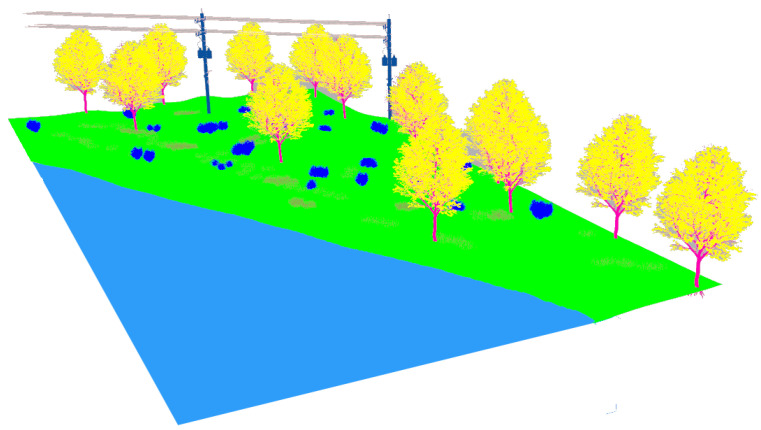
The plain visual model of the lake environment.

**Figure 15:**
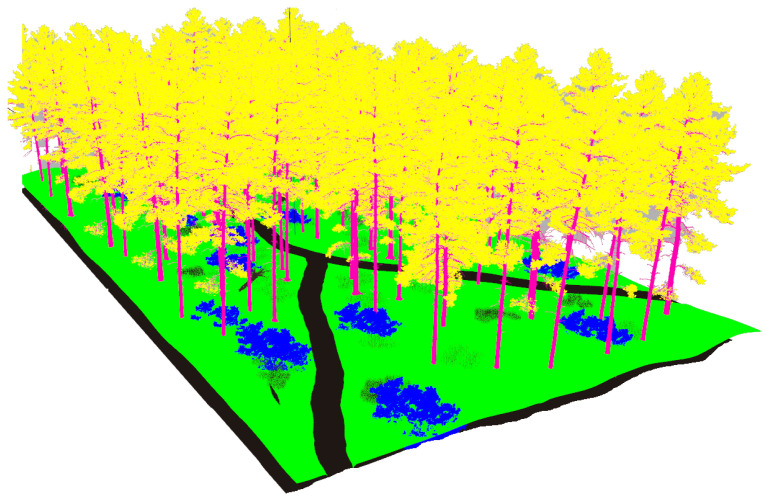
The plain visual model of the forest environment.

**Figure 16:**
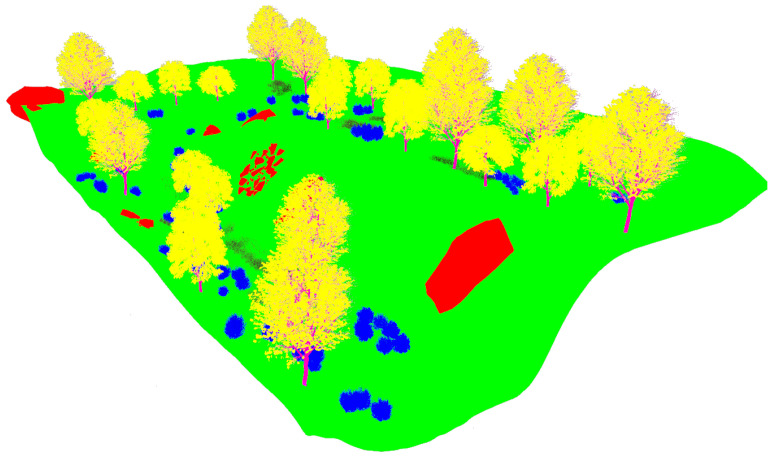
The plain visual model of the hillside environment.

The realistic visual models employ the naturalistic textures of each element provided by Gazebo including lighting conditions as shown in Figure 4, Figure 5, Figure 6 and Figure 7. The plain visual models are obtained by substituting these textures by the flat colors of Table 3 and by eliminating shadows (see Figure 13, Figure 14, Figure 15 and Figure 16).

The global pose of the UGV (i.e., the position and attitude of base_link) is extracted with a frequency of 25 Hz from the recorded bag file of the experiment and employed to place the robot in the environment. Then, static images from the left_camera and the right_camera frames are taken using both the realistic and the plain visual models, whose messages are inserted in the bag file synchronously.

**Figure 17:**
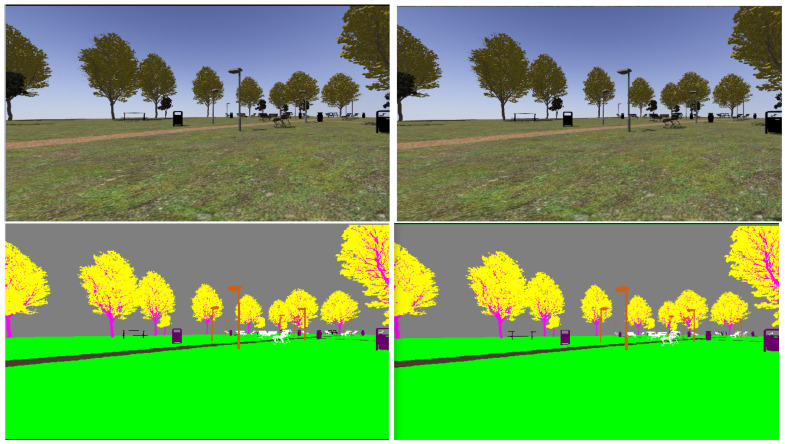
Realistic (**up**) and tagged (**down**) stereo image pairs (**left-right**) taken from the park environment.

**Figure 18:**
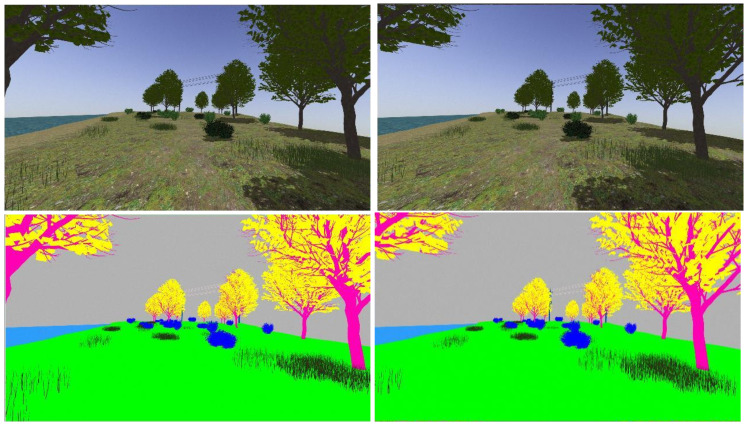
Realistic (**up**) and tagged (**down**) stereo image pairs (**left-right**) taken from the lake environment.

**Figure 19:**
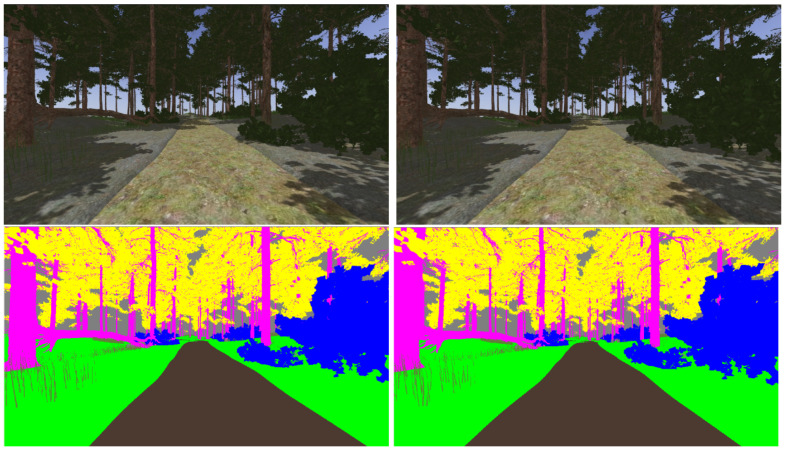
Realistic (**up**) and tagged (**down**) stereo image pairs (**left-right**) taken from the forest environment.

**Figure 20:**
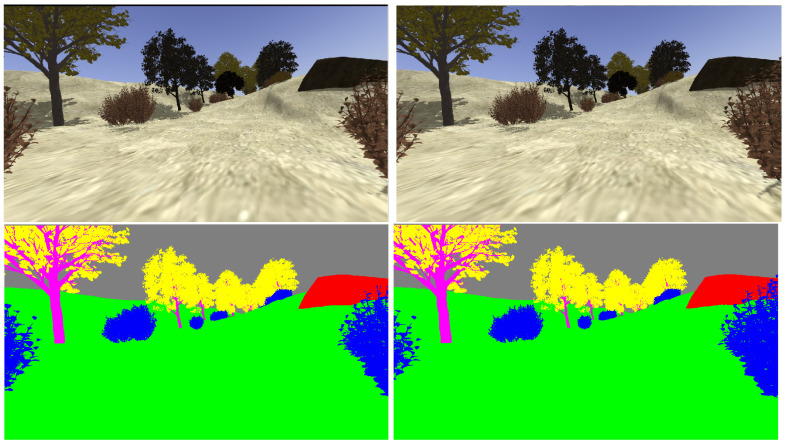
Realistic (**up**) and tagged (**down**) stereo image pairs (**left-right**) taken from the hillside environment.

Figure 17, Figure 18, Figure 19 and Figure 20 show a couple of realistic and flat-color images, as they have been captured from the same point of view in each environment. In this way, an exact correspondence at the pixel level can be achieved.

## 5. Dataset Description

The dataset has been divided into several bag files, each one corresponds to a different experiment. The number of sensor readings contained in each bag file along with the length and the time extend of the trajectories can be found in Table 4.

Table 5 shows the main messages produced by ROS topics that are recorded in the bag files. Each message contains a header with a time-stamp that indicates the exact moment when the message was sent during the simulation. The highest update rate of 1000 Hz corresponds to the ODE physics solver.

**Figure 21:**
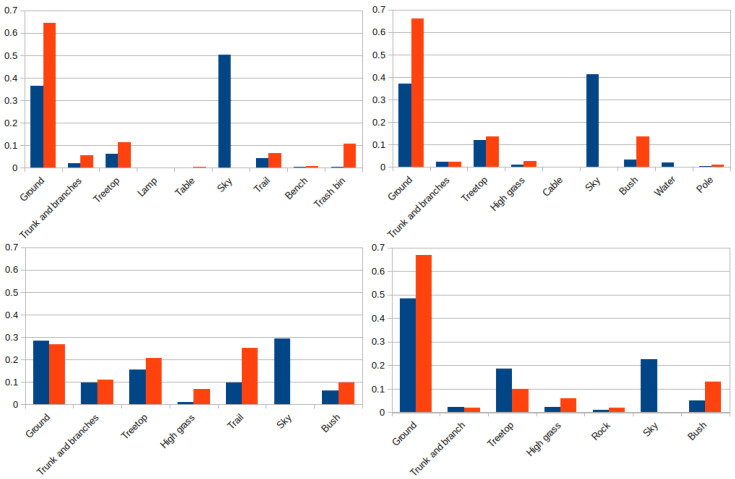
Label distribution for pixels (blue) and 3D points (red) in the park (**up-right**), lake (**up-left**), forest (**down-right**) and hill (**down-left**) environments.

Figure 21 shows the proportion, expressed on a per unit basis, of 3D points and pixels for every element from the two experiments of each environment. In the park and lake settings there are nine different elements, and only seven for the forest and hill environments. It can be observed that most of the pixels of the images are labeled as ground or sky, except for the forest, with a more even distribution between its elements. This similarly happens with the 3D laser points, where the vast majority of them belong to the ground with the exception of the forest setting.

The bag files of the experiments are provided in a lossless compressed Zip format. Their companion text files correspond to SHA256 checksums to ensure that the decompressed files are right.

**Figure 22:**
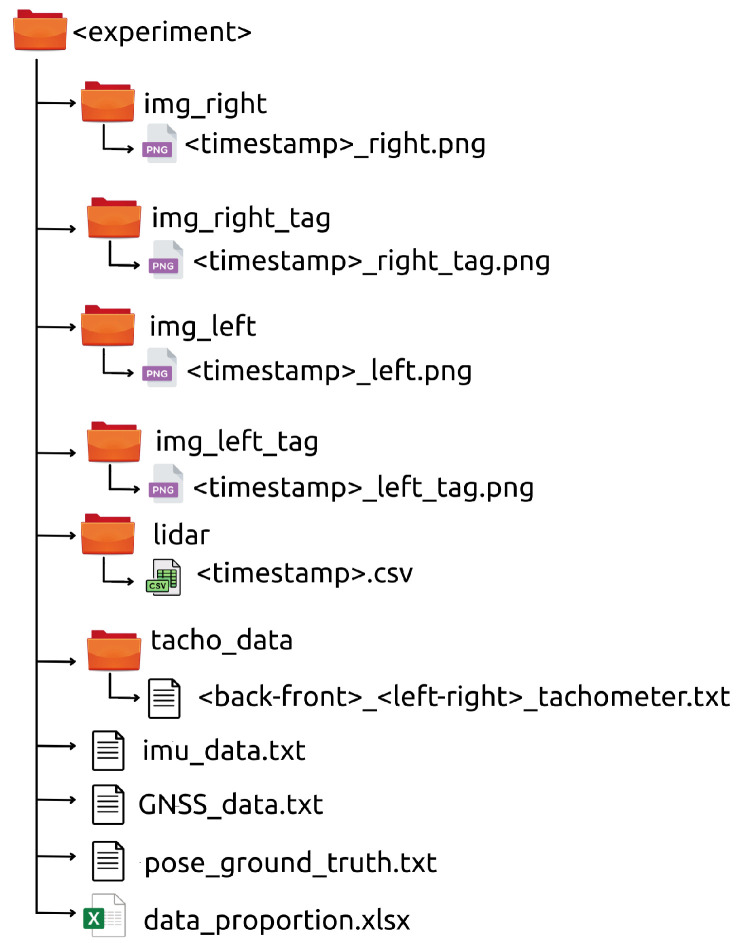
Human-readable contents of an experiment in the dataset.

Apart from the bags, the recorded data is also provided in a human-readable format (see Figure 22). In this way, every experiment includes the following files and folders:

img_left, img_right, img_left_tag, img_right_tag. These folders contain all the generated left and right (both realistic and tagged) images, respectively. The stereo images have been saved with the Portable Network Graphics (PNG) format, where its time-stamp is part of the filename.
lidar. This folder contains all the generated 3D point clouds. Every one has been saved in the Comma-Separated Values (CSV) format and with its time-stamp as its filename. Each line consists of the Cartesian coordinates with respect to the os1_lidar frame and the object reflectivity.
imu_data, GNSS_data. The IMU and GNSS data have been saved separately as text files, where each sensor reading is written in a new line that starts with its corresponding time-stamp.
tacho_data. The tachometer readings of each wheel are provided in four separated text files. Each line contains the time-stamp and the wheel speed in rad/s.
pose_ground_truth. This text file contains the pose of the UGV, given for its base_link frame, published by the /gazebo/model_states topic. Each line begins with a time-stamp, continues with Cartesian coordinates and ends with a unit-quaternion.
data_proportion. The exact label distribution among pixels and 3D points for the experiment are detailed in this Excel file.

### Additional Material

The auxiliary software tools and files that are required to perform the simulations have also been released on the dataset website. They have been tested with Ubuntu 18.04 Operating System and the full Melodic Morenia distribution of ROS (which includes Gazebo 9). Both are open-source projects freely available on their respective websites.

Two compressed files and a README text file are provided. The latter contains the instructions for installation and for usage with this setup. The contents of the first compressed file are as follows:

husky. A modified version of the Clearpath Husky stack that includes a customized version of the husky_gazebo package with the sensor setup described in the paper and the new package husky_tachometers with the plugin for wheel tachometers on Husky.
geonav_transform. This package is required to simplify the integration of GNSS data into the ROS localization and navigation workflows.
ouster_gazebo. The Ouster LiDAR model in Gazebo provided by the manufacturer.
natural_environments. The description of each environment in Gazebo and their launch files. This folder also includes the way-point navigation node and the script for annotating images.

On the other hand, the second compressed file contains all the 3D Gazebo models of the elements present in the four environments.

## 6. Conclusions

This paper has described a synthetic dataset obtained with Gazebo by closely mimicking the navigation of a ground mobile robot in natural environments. It has been developed through a process that involves modeling, simulation, data recording and semantic tagging.

Gazebo modeling includes four different environments and a Husky UGV equipped with tachometers, GNSS, IMU, stereo camera and LiDAR sensors. ROS programming has been employed to follow waypoints during Gazebo simulations.

Unique reflectively values and flat colors have been assigned to environmental elements to automatically generate 37,620 annotated 3D point clouds and 92,419 tagged stereo images both totally free of classification errors. The dataset, which also contains UGV ground-truth, has been made available as ROS bag files and in human-readable formats.

Possible applications of this dataset include UGV navigation benchmarking and supervised learning in natural environments. Furthermore, to easily adapt the dataset or to directly employ the simulations, all the required software has also been released on the dataset website.

Future work includes the introduction of different lighting conditions and dynamic elements such as aerial vehicles [39]. It is also of interest to train context-aware navigation to avoid non-traversable zones via repeated simulations using reinforcement learning before testing it on a real mobile robot.

## Figures and Tables

**Table 1:** Main specifications of the ZED-2 cameras and of the OS1-64 LiDAR.

	ZED-2	OS1-64
Field of view (horizontal × vertical)	$69{}^{\circ }\times 42{}^{\circ }$	$360{}^{\circ }\times 45{}^{\circ }$
Resolution (horizontal × vertical)	1280×720 pixels	512×64 3D points
Output rate	25 Hz	10 Hz

**Table 2:** Relative poses of all the robot reference frames with respect to base_link.

Coordinate System	*x*	*y*	*z*	Roll	Pitch	Yaw
	(m)	(m)	(m)	(°)	(°)	(°)
GNSS_link	0.10	0	0.890	0	0	0
os1_lidar	0.09	0	0.826	0	0	0
right_camera	0.15	−0.06	0.720	0	90	0
left_camera	0.15	0.06	0.720	0	90	0
imu_link	0.19	0	0.149	0	−90	180
front_right_wheel	0.256	−0.2854	0.03282	0	0	0
rear_right_wheel	−0.256	−0.2854	0.03282	0	0	0
front_left_wheel	0.256	0.2854	0.03282	0	0	0
rear_left_wheel	−0.256	0.2854	0.03282	0	0	0
base_footprint	0	0	−0.13228	0	0	0

**Table 3:** RGB color tags and reflectivity values assigned to the elements present in the environments.

Element	Flat Color		Reflectivity
Ground	(0, 255, 0)	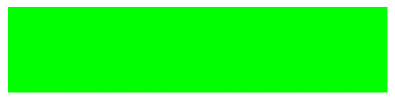	1
Trunk and branch	(255, 0, 255)	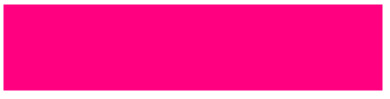	2
Treetop	(255, 255, 0)	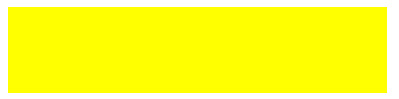	3
Bush	(0, 0, 255)	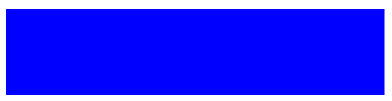	4
Rock	(255, 0, 0)	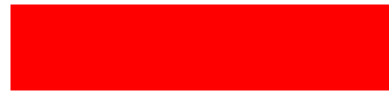	5
High grass	(97, 127, 56)	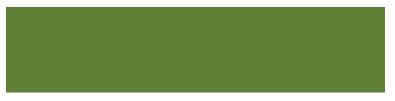	6
Sky	(127, 127, 127)	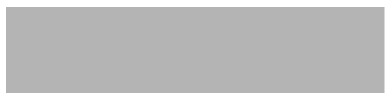	-
Trail	(76, 57, 48)	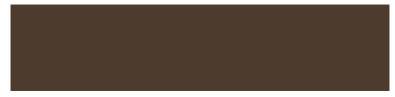	7
Lamp post	(204, 97, 20)	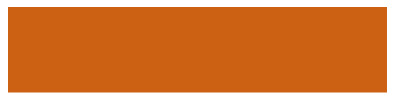	8
Trash bin	(102, 0, 102)	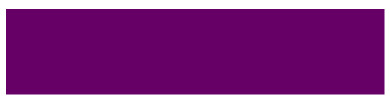	9
Table	(0, 0, 0)	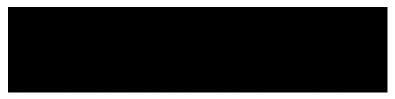	10
Water	(33, 112, 178)	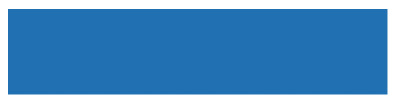	-
Bench	(255, 255, 255)	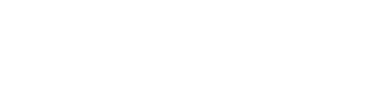	11
Post	(61, 59, 112)	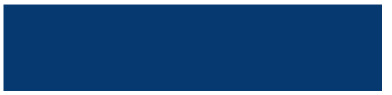	12
Cable	(255, 153, 153)	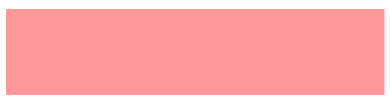	13

**Table 4:** Numerical information of the eight bag files.

Bag File	3D Point	Stereo	GNSS	IMU	Length	Duration
	Clouds	Images	Readings	Readings	(m)	(s)
Park 1	2576	6464	10,344	12,650	76.08	253
Park 2	7089	15,469	25,471	35,900	217.51	718
Lake 1	6216	15,562	24,900	31,100	186.85	622
Lake 2	6343	15,858	25,375	31,700	190.45	634
Forest 1	2689	6723	10,758	13,450	80.52	269
Forest 2	2451	6125	9802	12,250	73.38	245
Hillside 1	5145	13,162	20,583	25,700	153.10	514
Hillside 2	5111	13,056	20,444	25,550	159.34	511

**Table 5:** Main contents of a bag file of the synthetic dataset.

ROS Topic	Rate	Brief Description
{message}	(Hz)	
gazebo/model_states {gazebo/ModelStates}	1000	The pose of all objects in the environment, including Husky, with respect to the global coordinate frame of Gazebo.
imu/data {sensor_msgs/Imu}	50	3D attitude, linear acceleration and angular velocities of the UGV measured by its IMU.
navsat/fix {sensor_msgs/NavSatFix}	2	Geodetic coordinates (λ, ϕ and *h*) of the GNSS antenna.
os1_cloud_node/points {sensor_msgs/PointCloud2}	10	A 3D point cloud generated by the LiDAR, including its intensity measurements.
/gazebo_client/front_left_speed {std_msgs/Float32}	10	Angular speed of the front left wheel in rad/s.
/gazebo_client/front_right_speed {std_msgs/Float32}	10	Angular speed of the front right wheel in rad/s.
/gazebo_client/rear_left_speed {std_msgs/Float32}	10	Angular speed of the rear left wheel in rad/s.
/gazebo_client/rear_right_speed {std_msgs/Float32}	10	Angular speed of the rear right wheel in rad/s.
stereo/camera/left/real/compressed {sensor_msgs/CompressedImage}	25	A compressed realistic image of the left camera.
stereo/camera/left/tag/img_raw {sensor_msgs/Image}	25	An annotated image of the left camera.
stereo/camera/right/real/compressed {sensor_msgs/CompressedImage}	25	A compressed realistic image of the right camera.
stereo/camera/right/tag/img_raw {sensor_msgs/Image}	25	An annotated image of the right camera.

## Data Availability

The dataset presented in this study is openly available at: https://www.uma.es/robotics-and-mechatronics/info/132852/negs-ugv-dataset (accessed on 22 July 2022).

## Abbreviations

3D	Three-Dimensional
CSV	Comma-Separated Values
GNSS	Global Navigation Satellite System
IMU	Inertial Measurement Unit
LiDAR	Light Detection and Ranging
ODE	Open Dynamics Engine
PNG	Portable Network Graphics
ROS	Robot Operating System
SAR	Search and Rescue
SLAM	Simultaneous Localization and Mapping
UGV	Unmanned Ground Vehicle
